# Ordering of Primary Carbonitrides in an Austenitic Steel Revealed by Transmission Electron Microscopy and Atom Probe Tomography

**DOI:** 10.3390/ma11112321

**Published:** 2018-11-19

**Authors:** Ping Ou, Zhiming Li

**Affiliations:** 1 School of Materials Science and Engineering, Jiangxi University of Science and Technology, Ganzhou 341000, China; opyp@163.com; 2 Max-Planck-Institut für Eisenforschung, Max-Planck-Str. 1, 40237 Düsseldorf, Germany; 3 School of Materials Science and Engineering, Central South University, Changsha 410083, China

**Keywords:** carbonitride, ordering, austenitic steel, transmission electron microscopy, atom probe tomography

## Abstract

We reveal for the first time an ordering phenomenon of a type of carbonitrides in a Super304H austenitic steel via the techniques of transmission electron microscopy and atom probe tomography. Solution-treated Super304H austenitic steel samples containing a few primary carbonitrides were aged at 923 K for 5000 h. The results show that the primary carbonitrides in the Super304H steel are non-stoichiometric compounds enriched in Nb and with a NaCl-type structure. The crystal structure of the Nb-rich carbonitrides is in a disordered state in the solution-treated steel, while in an ordered state in the aged steel. This observation suggests the occurrence of a disorder–order structural transition in the primary carbonitrides during long-term aging. We found that such a disorder–order structural transition is accompanied by carbon diffusion from the primary carbonitrides into the austenitic matrix. The ordering phenomenon is discussed based on the long-range ordering of structure vacancies in the non-metal lattice of the superstructure, providing new insights for the understanding of ordering in non-stoichiometric compounds.

## 1. Introduction

**Figure 1:**
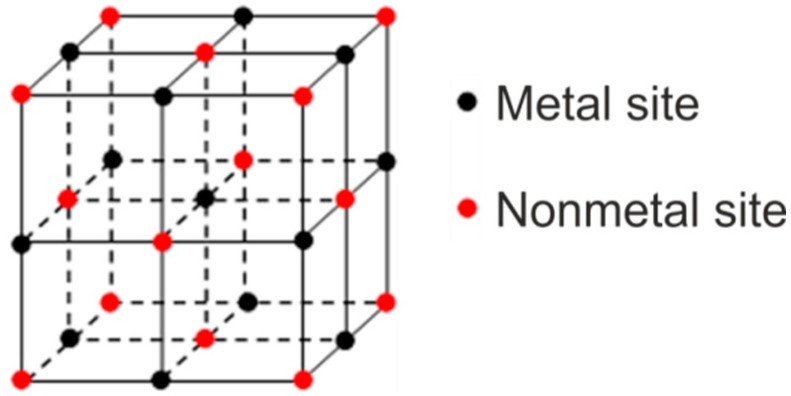
Schematic sketch showing the lattice structure of the MX phase.

Super304H austenitic steel, which is based on 18Cr-8Ni stainless steel and alloyed with about 3 wt.% Cu and a small amount of Nb (<1 wt.%) and N (<0.2 wt.%), is widely used in ultra-super critical (USC) power plants due to its excellent high-temperature performance, especially high-temperature creep resistance [1,2,3]. The outstanding creep strength of Super304H steel arises from the precipitation of nanosized Cu-rich and MX (Nb(C,N)) phases in the austenitic matrix when serving at high temperatures [1,2]. In addition to nanosized MX precipitates, Nb can also exist in large Nb-rich particles of micrometer sizes, which are the primary carbides or carbonitrides in Super304H steel and usually considered to be harmful for the mechanical properties of the steel. The formation of MX phase occurs when strong carbide/nitride former elements such as Ti, Nb and V are added in steels [4]. As shown in Figure 1, the MX phase has a NaCl-type structure that can be viewed as two interpenetrating face-centered cubic (fcc) lattices. The octahedral interstices of fcc metal sublattice are occupied by non-metal atoms X, which in turn form an fcc non-metal sublattice. In some cases, non-metal atoms X only occupy a part of interstices depending on their relative content *y*, which can lead to the formation of non-stoichiometric compounds MX*_y_*. Accordingly, the unoccupied interstices are referred to as the structural vacancies. These non-stoichiometric compounds are referred to as substances with structural vacancies and homogeneity intervals [5,6]. A homogeneity interval in a non-stoichiometric compound refers to a concentration region within which the crystal structure of the compound remains unchanged if the concentrations of the constituent elements are changed [7].

There are two different variants of non-stoichiometric compounds MX*_y_*, i.e., disordered and ordered. For instance, in disordered binary MX*_y_* compounds, vacancies randomly locate at the lattice sites of the basic structure without long-range ordering. Accordingly, the ordered binary MX*_y_* compounds are formed by a redistribution of vacancies over the structural sites so that one of the sublattices is further divided into two sublattices of atoms and vacancies, respectively [5,6,7,8,9,10]. In the Nb–C system, the disordered NbC*_y_* carbide with a NaCl-type structure is a type of non-stoichiometric compound and usually shows an abroad homogeneity interval [7]. The non-stoichiometric NbC*_y_* carbide is generally in a disordered state but its ordering has also been found before. It has been reported that annealing-disordered NbC*_y_* carbide with a homogeneity interval 0.81 ≤ *y* ≤ 0.88 at a temperature below 1300 K can induce the formation of ordered Nb_6_C_5_ phase [7,11]. A trigonal structure analogous to V_6_C_5_ had been proposed originally for describing the observed superstructure of the Nb_6_C_5_ phase [12,13,14,15,16,17,18]. However, later work has suggested it to be monoclinic superstructure [19]. Also, the Nb–C phase diagram proposed in the literature [7] shows that transformation of the Nb_6_C_5_ phase to the ordered Nb_3_C_2_ phase with a narrow homogeneity interval can occur at a temperature below 900 K under thermodynamic equilibrium conditions but it has not been experimentally observed in NbC*_y_* carbide so far.

Under certain conditions, the N atoms can also occupy the octahedral interstices of the metallic sublattice in NbC*_y_* carbide, which leads to the formation of Nb(C,N)*_y_* (0 *< y* ≤ 1) carbonitrides. The Nb(C,N)*_y_* carbonitride has been reported to be in a disordered state, whereas its ordering has not been found so far. In the present study, we reveal a new ordering phenomenon of carbonitrides in a Super304H austenitic steel upon long-term (e.g., 5000 h) aging. Accordingly, we investigated the crystal structure and chemical composition of the Nb(C,N)*_y_* carbonitrides in Super304H steels by transmission electron microscopy (TEM) and atom probe tomography (APT), respectively. Two types of Super304H steel samples, i.e., the solution-treated and long-term (e.g., 5000 h) aged have been considered. The ordering phenomenon in the Nb(C,N)*_y_* carbonitrides is then revealed and the related mechanisms are discussed in the following.

## 2. Experimental Procedures

The solution-treated Super304H austenitic steel was used as starting material, which was heat-treated at 1433 K for 15 min followed by water cooling. The chemical composition of the Super304H steel is given in Table 1. Some of the solution-treated samples were aged at 923 K for 5000 h followed by air cooling. The microstructure of both the solution-treated and long-term aged Super304H specimens were examined by means of a JSM-7600F field emission scanning electron microscope (SEM) equipped with energy-dispersive X-ray spectroscopy (EDS) system. The samples for SEM observations were prepared by mechanical grinding followed by fine polishing using an oxide suspension (OPS) with silica particle sizes around 50 nm. The microstructures of the solution- and aging-treated steels were further analyzed using TEM. The samples for TEM observations were prepared by twin-jet electropolishing in a solution consisting of 5 vol.% perchloric acid and 95 vol.% ethanol at about 243 K and 60 V. TEM observations were conducted on a JEOL 2100F machine operating at 200 kV. The needle-like APT specimens of austenitic matrix and carbonitrides were prepared using a dual-beam focused-ion beam (FIB) system (FEI Helios Nano-Lab 600i) through a site-specific lift-out procedure [20]. For the preparation of carbonitride tips, a thin layer of Pt was deposited on the carbonitride of interest before the start of lift-out. Annular FIB milling was performed using a low acceleration voltage of 5 kV at final polishing to keep Ga implantation at a negligible level. APT analysis was conducted using a LEAP 3000X HR system (Cameca Instruments) in voltage-pulsing mode at 200 kHz pulse repetition rate, 0.005 atom/pulse detection rate, 15% pulse fraction, and 70 K. Six successful measurements for each sample condition were performed and evaluated. The APT data were evaluated using the IVAS software from Cameca Instruments (version 3.6.10).

## 3. Results

### 3.1. Carbonitrides in Solution-Treated Steel

**Figure 2:**
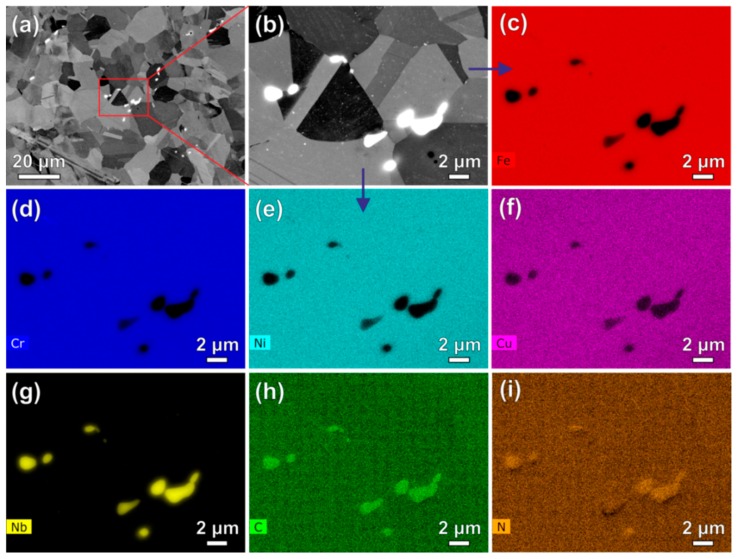
Back-scattered electron (BSE) images of the solution-treated Super304H steel sample with relatively low (**a**) and high (**b**) magnifications. (**c**–**i**) are the energy-dispersive X-ray spectroscopy (EDS) maps of elements Fe, Cr, Ni, Cu, Nb, C and N, respectively, corresponding to the identical sample region in (**b**).

Figure 2a,b shows the back-scattered electron (BSE) images of the solution-treated Super304H steel with relatively low and high magnifications, respectively. Equiaxed austenitic grains show an average size of ~15 µm. A small amount of primary carbonitrides (~0.5 vol.%) with sizes ranging from hundreds of nanometers to several micrometers locate in the vicinity of the austenitic grain boundaries. The different compositions in the austenitic matrix and primary carbonitrides lead to their varying contrast observed from the BSE images. To reveal the corresponding elemental distributions, EDS maps of elements Fe, Cr, Ni, Cu, Nb, C and N with an identical sample region in Figure 2b are shown in Figure 2c–i, respectively. It is confirmed that the primary carbonitrides are enriched in Nb, C and N while depleted in Fe, Cr, Ni and Cu compared to the austenitic matrix.

**Figure 3:**
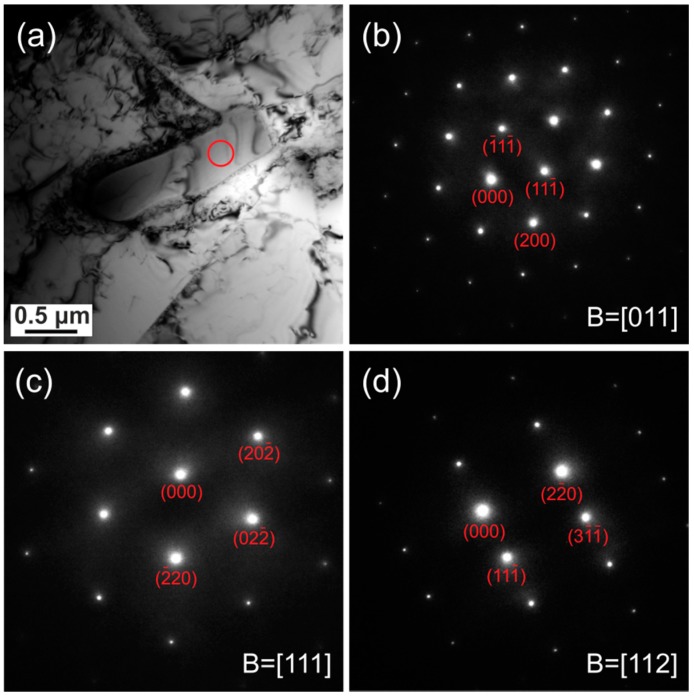
(**a**) Bright field transmission electron microscope (TEM) image of a typical primary carbonitride in the solution-treated Super304H steel. (**b–d**) are selected area electron diffraction (SAED) patterns of the carbonitrides taken along [011], [111] and [112] zone axis, respectively.

TEM and selected area electron diffraction (SAED) were employed to characterize the primary carbonitrides in the Super304H steel. Figure 3a shows a bright field TEM image of a typical primary carbonitride in the solution-treated Super304H steel. The SAED patterns collected from the carbonitrides are presented in Figure 3b–d, which were taken along [110], [111] and [112] zone axes, respectively. The SAED patterns were taken from various specimen tilts over several different carbonitride particles given the aim of a comprehensive characterization. These SAED patterns confirm that the primary carbonitrides have a NaCl-type structure. Note that extra diffraction spots do not exist in the SAED patterns, indicating that the crystal structure of the Nb-rich carbonitrides is in disordered state in the solution-treated Super304H steel. The lattice constant of the disordered Nb-rich carbonitrides is estimated to be ~0.460 nm from the SAED patterns, which is very close to the lattice constants reported for NbC (*a* = 0.447 nm) [21] and NbN (*a* = 0.439 nm) [21].

**Figure 4:**
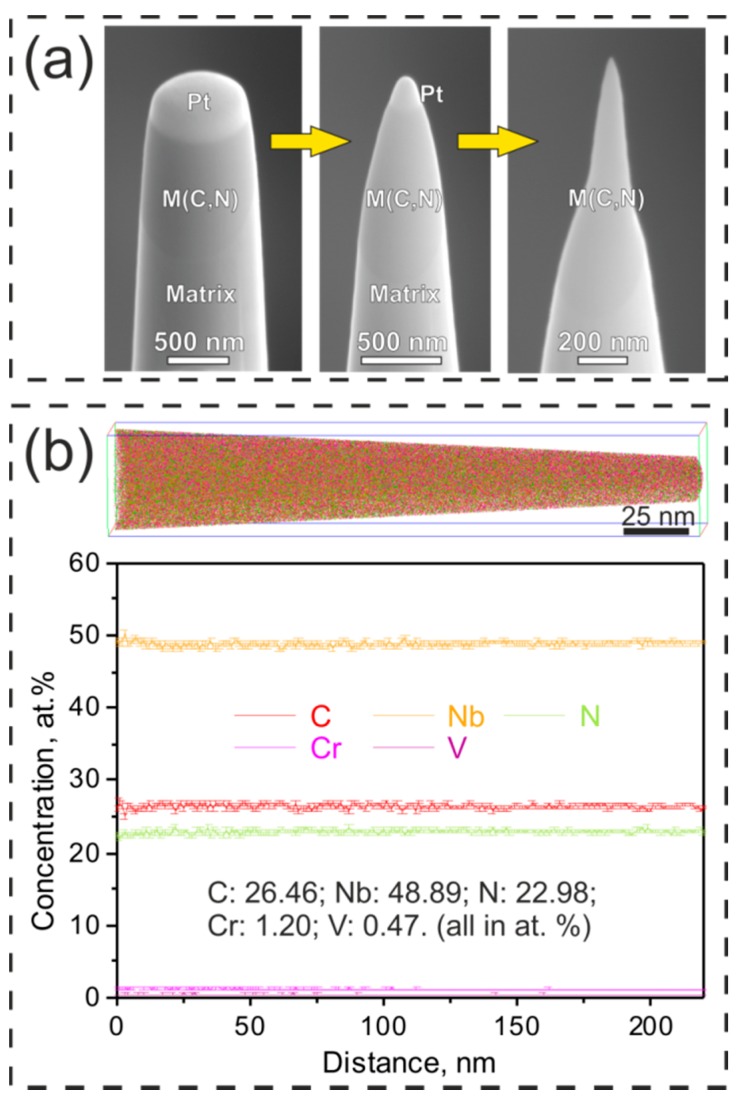
(**a**) Secondary electron (SE) images showing the preparation process of the atom probe tomography (APT) tips of carbonitrides by focus-ion beam (FIB) technique. The Pt layer was employed to mark and protect the target carbonitride during lift-out process. (**b**) Three-dimensional reconstruction and one-dimensional compositional profiles of all elements in a representative APT tip taken from a carbonitride in the solution-treated Super304H steel.

To examine the exact concentrations of elements in the primary carbonitrides in the Super304H steel, APT analysis was conducted and needle-shaped specimens of the carbonitrides were prepared by a site-specific lift-out procedure using FIB technique [20]. The secondary electron (SE) images in Figure 4a shows the preparation process of a typical needle-shaped APT specimen. The Pt layer was employed to mark and protect the target carbonitride during the lift-out process. The three-dimensional reconstruction of all elements in a representative APT tip taken from a carbonitride in the solution-treated Super304H steel (Figure 4b) shows that all the elements including C, N, Nb, Cr and V are uniformly distributed in the carbonitride. One-dimensional compositional profiles along the length direction of the tip are shown in Figure 4b, and the results clearly confirm that there is no apparent fluctuation of elemental fractions in the probed specimen. Moreover, the tip of the primary carbonitride in the solution-treated Super304H steel has an overall composition of (Nb_48.89_Cr_1.20_V_0.47_)(C_26.46_N_22.98_) according to the APT analysis (Figure 4b). This suggests that besides the Nb as the main metal elements, there are also a small amount of metal lattice sites occupied by Cr and V in the disordered carbonitride. Furthermore, the average ratio of metal to non-metal atoms is around 1/0.978 (±0.005) based on the analysis of more than five carbonitride particles, indicating that the disordered carbonitrides in the solution-treated Super304H steel are non-stoichiometric and a number of structural vacancies are located at the non-metal lattice sites.

### 3.2. Carbonitrides in Long-Term Aged Steel

**Figure 5:**
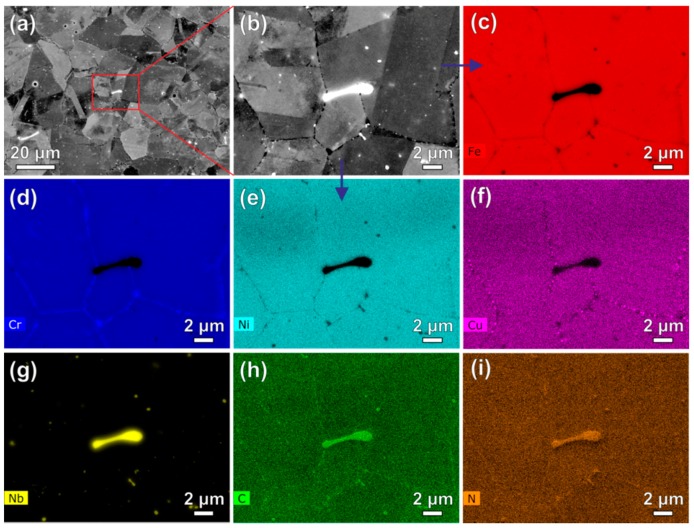
Back-scattered electron (BSE) images of the long-term (5000 h at 923 K) aged Super304H steel sample with relatively low (**a**) and high (**b**) magnifications. (**c–i**) are the EDS maps of elements Fe, Cr, Ni, Cu, Nb, C and N, respectively, corresponding to the identical sample region in (**b**).

Figure 5a,b shows the BSE images of the microstructure in the long-term (5000 h at 923 K) aged Super304H steel. The average size of the equiaxed austenitic grains (~15 µm) in the long-term aged sample is similar to that in the solution-treated sample. The primary carbonitrides are clearly presented in white due to the sharp phase contrast between the austenitic matrix and the primary carbonitride particles. Figure 5c–i show the EDS maps of elements Fe, Cr, Ni, Cu, Nb, C and N with an identical sample region in Figure 5b. Analogous to that observed in the solution-treated case, the primary carbonitride particles in the long-term aged Super304H steel are also enriched in Nb, C and N while depleted in Fe, Cr, Ni and Cu. Additionally, grain boundary phase enriched in Cr and C while depleted in Fe and Ni can also be observed from the EDS maps (Figure 5c–i). According to the previous study [1,2,22,23], such a grain boundary phase in Super304H steel is M_23_C_6_ carbide formed during the long-term aging process. However, we focus on the primary carbonitrides and, therefore, the characterization of M_23_C_6_ carbides formed during aging is beyond the scope of the present study.

**Figure 6:**
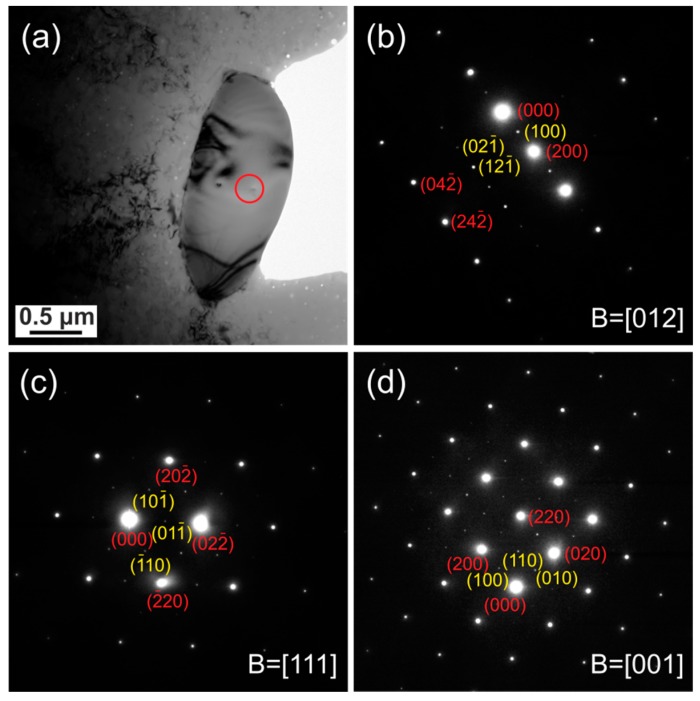
(**a**) Bright field TEM image of a typical primary carbonitride in the long-term aged Super304H steel. (**b–d**) are selected area electron diffraction (SAED) patterns of the carbonitrides taken along [012], [111] and [001] zone axis, respectively.

Figure 6a shows a bright field TEM image of a typical primary carbonitride in the aged Super304H steel. The SAED patterns of the carbonitrides taken along [012], [111] and [001] zone axes are shown in Figure 6b–d, respectively. These SAED patterns confirm that the primary carbonitride in the aged Super304H steel also has a NaCl-type structure. Interestingly, all the SAED patterns in Figure 6b–d show the extra {h/2 k/2 l/2} superlattice diffraction spots, indicating that the crystal structure of the Nb-rich carbonitride is in an ordered state in the aged Super304H steel. According to the SAED patterns, the lattice constant of the ordered Nb-rich carbonitrides was estimated to be ~0.467 nm, which is similar to that of the disordered Nb-rich carbonitrides.

**Figure 7:**
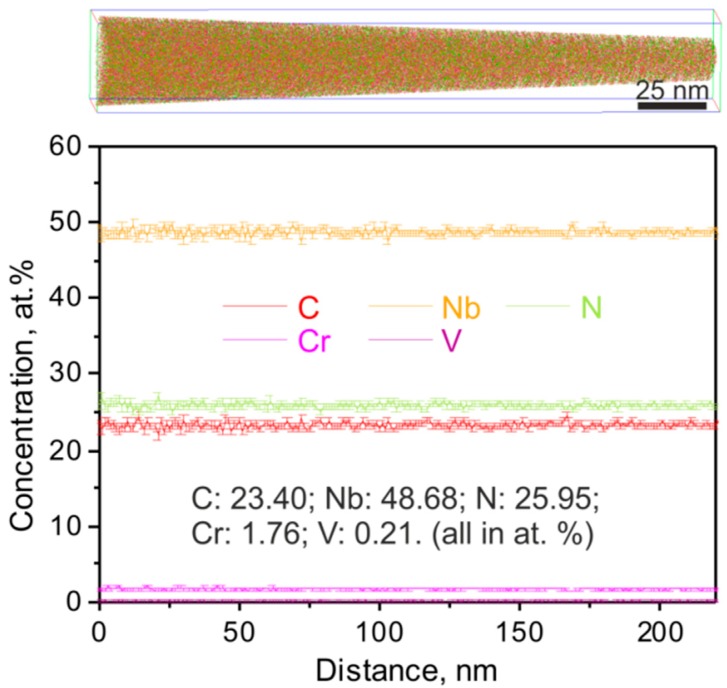
Three-dimensional reconstruction and one-dimensional compositional profiles of all elements in a representative APT tip taken from a carbonitride in the long-term aged Super304H steel.

Figure 7 shows the three-dimensional reconstruction and one-dimensional compositional profiles of all elements in a representative APT taken from a carbonitride in the long-term aged Super304H steel. Basically, all the elements are still uniformly distributed in the carbonitrides after the long-term aging. One-dimensional concentration profiles of all the elements indicate that the tip of the carbonitride has an overall composition of (Nb_48.68_Cr_1.76_V_0.21_)(C_23.40_N_25.95_). The average ratio of metal to non-metal atoms is around 1/0.974 (±0.007) in the carbonitride after aging, indicating that the ordered carbonitrides in the aged Super304H steel are also non-stoichiometric. Furthermore, the structural vacancies located at the non-metal lattice sites in the ordered carbonitrides in the long-term aged sample are slightly higher than those in disordered carbonitrides in the solution-treated sample. It is important to note that the carbon concentration in the ordered carbonitrides in the long-term aged steel (23.40 at.%) is lower than that in the disordered carbonitrides in the solution-treated steel (26.46 at.%). However, the nitrogen concentration was increased from 22.98 at.% to 25.95 at.% after the long-term aging. This observation indicates that the carbon atoms are easier to diffuse than the nitrogen atoms from primary carbonitride to austenitic matrix during aging. The mechanism underlying this phenomenon will be discussed in the following section.

## 4. Discussion

### 4.1. Carbon Diffusion

**Figure 8:**
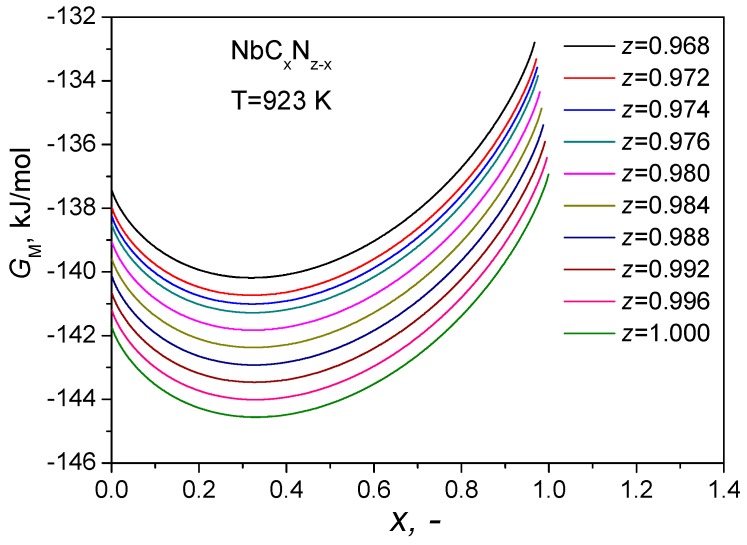
Dependence of the Gibbs energy (*G_M_*) of the NbC*_x_*N(_*z*−*x*_) carbonitrides on the fraction of carbon sites (x) under different values of the total fraction of non-metal sites (z) at the aging temperature (923 K).

To explain the variations of carbon and nitrogen concentrations in the primary carbonitride in the Super304H steel after long-term aging, we first discuss the lattice stabilities of the primary carbonitride at different heat-treatment conditions (e.g., solution-treated and long-term aged) by evaluating the corresponding Gibbs energy changes. This is based on the fact that the Gibbs energy of a compound depends on its composition at a given temperature [24,25]. From the above APT results, the concentrations of Cr and V in the carbonitride in both the solution-treated and long-term aged samples are very low (<2 at.% in total) and, therefore, the effects of Cr and V on Gibbs energy of the carbonitrides were ignored in our following thermodynamic analysis. Therefore, all the metal lattice sites of carbonitrides are considered to be occupied by Nb atoms, and the overall compositions of carbonitrides in the solution-treated and long-term aged samples can be represented simply as NbC_0.523_N_0.454_ and NbC_0.462_N_0.512_, respectively. Although the primary carbonitrides in the long-term aged sample have a slightly higher amount of structural vacancies compared to that in the solution-treated sample, they both have a NaCl-type structure. This implies that there is a homogeneity interval in such a NaCl-type structure of the primary carbonitrides, which is experimentally revealed in the present study as the first time. Based on our results, the homogeneity interval of primary carbonitrides in the Super304H steel can be estimated to be in a range from 0.968 to 1. The primary carbonitrides in the Super304H steel is represented by NbC*_x_*N_(*z*−*x*)_, where x (0≤x≤z) is the fraction of carbon sites and z (0.968≤z≤1) is the total fraction of non-metal sites. Accordingly, (*z*−*x*) is the fraction of nitrogen sites. As mentioned, the NaCl-type structure can be regarded as composed of two sublattices, i.e., fcc metal sublattice and fcc non-metal sublattice. According to the two-sublattice compound energy model [24,25], the Gibbs energy of the primary carbonitride in the Super304H steel, *G_M_*, can be expressed as:

(1) $$G_{M}=xG_{NbC}^{o}+(z-x)G_{NbN}^{o}+RT(x\ln x+(z-x)\ln(z-x))+x(z-x)L_{C,N}^{Nb}\;$$

where *R* is the gas constant, *T* is the absolute temperature, $L_{C,N}^{Nb}$ = 1.87 kJ/mol is the interaction parameter of Nb, C and N in the carbonitrides with a NaCl-type structure [26], $G_{NbC}^{o}$ and $G_{NbN}^{o}$ are the Gibbs energies of NbC and NbN, which are taken as −136.88 kJ/mol [27] and −141.69 kJ/mol [26] at the temperature of 923 K, respectively. Accordingly, the relationship between the Gibbs energy (*G_M_*) and the fraction of carbon sites (*x*) under different values of total fraction of non-metal sites (*z*) in the NbC*_x_*N_(*z*−*x*)_ carbonitrides at the aging temperature (923 K) was calculated and the results are plotted in Figure 8. Under different values of *z*, the Gibbs energy first decreases with increasing the fraction of carbon sites (*x*) and then increases after reaching the minimum value. The fraction of carbon sites (*x*) corresponding to the minimum Gibbs energy under different values of *z* can be estimated using Equation (1). The results suggest that the value of *x* for the minimum Gibbs energies ranges from 0.315 to 0.325 under different values of *z* from 0.968 to 1.

In the present study, the fraction of carbon sites (*x*) in the solution-treated sample is measured to be around 0.523, which is significantly higher than that corresponding to the minimum Gibbs energy at the aging temperature (923 K). This suggests that the primary carbonitrides have a relatively high Gibbs energy and hence are metastable at the aging temperature. On the other hand, the austenitic matrix of Super304H steel is a supersaturated solid solution after solution treatment, which is also metastable at 923 K. In fact, the primary carbonitrides observed in the solution-treated Super304H steel are residual carbonitrides that are not completely dissolved in the austenitic matrix during solution treatment. The dissolution process of carbonitride during heat treatment can be described using a local equilibrium model proposed by Hillert et al. [28]. According to this theory, the local equilibrium is established at the phase interface between austenitic matrix and primary carbonitride. The previous studies showed that discontinuous chains of M_23_C_6_ carbides with submicron sizes can be precipitated at grain boundaries and the MX (Nb(C,N)) precipitates with a nanosized diameter can be formed in grain interiors in the Super304H steel after long-term aging [1,2,22,23]. The precipitations of these different types of submicron carbides and nano-carbonitrides in the austenitic steel results in the reduction of carbon and nitrogen contents in the austenitic matrix, which certainly leads to the breakdown of the local equilibrium at the phase interface between the austenitic matrix and the primary carbonitride. As a result, the diffusion of non-metal atoms between the austenitic matrix and the primary carbonitride can be promoted during long-term aging. However, as shown in Figure 8, the reduction of nitrogen content in the NbC*_x_*N_(*z*−*x*)_ carbonitrides would cause an increase of Gibbs energy and hence reduce the lattice stability of the carbonitrides. Therefore, the carbon atoms are easier to diffuse than the nitrogen atoms from primary carbonitride to austenitic matrix during long-term aging.

It should be noted that the above calculations of Gibbs energy assume a disordered state of the primary carbonitrides. Although in principle the Gibbs energy of the ordered carbonitrides can also be evaluated by the two-sublattice compound energy model [24,25], the relevant thermodynamic parameters are absent at present. Based on the experimental results that the carbon atoms are easier to diffuse than the nitrogen atoms from primary carbonitride to austenitic matrix during the long-term aging, we speculate that the changes of Gibbs energy of the ordered NbC*_x_*N_(*z*−*x*)_ carbonitride should follow the same trend as that of the disordered carbonitrides.

### 4.2. Ordering of the Primary Carbonitrides

According to the TEM analysis, there is a disorder–order structural transition occurred in the primary carbonitrides in the Super304H steel during long-term aging. This is accompanied by the carbon diffusion from the primary carbonitrides into the austenitic matrix as revealed by APT analysis. From thermodynamic considerations, the crystal structure of a non-stoichiometric compound is energetically favored to be in ordered state at low temperatures, whereas a disordered state is more likely to present at high temperatures if the entropic contribution to the Gibbs energy is large enough [5,6,7,8,9,10]. Thus, the crystal structure of the disordered carbonitrides is metastable at the aging temperature (923 K), which results in the transition from the disordered to the ordered state during long-term aging. It has been established that ordering of the non-stoichiometric binary interstitial compounds MX*_y_* can take place via a redistribution of interstitial non-metal atoms and structure vacancies which are viewed as the components of a binary substitutional solution that is formed in the non-metal lattice under certain conditions [5,6,7,8,9,10]. As a result, the long-range ordering of vacancies can be formed in the non-metal lattice of the non-stoichiometric compounds.

For ordering mechanism in non-stoichiometric compounds such as M(C,N)*_y_*, it was proposed in one of the concepts that the sites of one of the superstructure sublattices would be filled preferentially with non-metal atoms of the first sort (e.g., C), while the sites of the other would be filled preferentially with non-metal atoms of the second sort (e.g., N) [7,11]. However, Gusev et al. [29,30] later investigated the ordering of non-stoichiometric vanadium carbonitrides V(C,N)_0.85_ with a V_6_C_5_ structure by X-ray diffraction and they proposed that the C and N atoms in the non-metal sublattice of these superstructures form only one sublattice, in which the C and N atoms are arranged in a random fashion, and structural vacancies form another sublattice. Up to now, few reports are dedicated to the ordering in transition-metal carbonitrides (M(C,N)*_y_*), and a unified understanding of the ordering process in non-stoichiometric carbonitrides is still absent so far.

In the present study, the crystal structure of the primary carbonitrides in the solution-treated Super304H steel is in disordered state, where the interstitial non-metal atoms and structure vacancies distribute randomly. As discussed in Section 4.1, the primary carbonitrides are in a non-equilibrium state at the aging temperature (923 K), and carbon atoms are thermodynamically driven to diffuse from the primary carbonitrides to the austenitic matrix during aging. Even if a redistribution of carbon and nitrogen atoms could occur to form a superstructure in the non-metal lattice, such a superstructure would be destroyed immediately due to the diffusion of carbon atoms from the primary carbonitrides to the austenitic matrix. Therefore, the ordering of the carbonitrides is more likely to be caused by the long-range ordering of structure vacancies in the non-metal lattice during aging. Accordingly, a redistribution of interstitial atoms (C and N) and structure vacancies over the non-metal lattice takes place so that the non-metal lattice is divided into an fcc sublattice of interstitial atoms and an fcc sublattice of structure vacancies inside the carbonitrides during long-term aging.

Non-stoichiometry occurs widely in solid-state compounds and is most pronounced in non-stoichiometric compounds, such as cubic carbides, nitrides and oxides of transition metals and related materials. From crystallographic considerations, the superstructure types corresponding to stoichiometric compositions, such as M_2_X, M_3_X_2_, M_4_X_3_, M_6_X_5_ and M_8_X_7_, can be formed when the vacancy concentration reaches a certain level [7,31,32]. Up to now, most of the above superstructures have been experimentally confirmed in the non-stoichiometric compounds [7,11]. Particularly, the M_8_X_7_ cubic superstructure has been found in ordered V_8_C_7_ [7,11] and V(C,N)_0.91_ [33], corresponding to the ordering phenomenon with the lowest structure vacancy concentration (~0.09 at.%) observed so far. However, it is still unclear whether the ordering phenomenon in the MX*_y_* compounds can occur under an even lower vacancy concentration. In the present study, we revealed for the first time that a new ordered Nb-rich carbonitrides with a NaCl-type structure and a relatively low structure vacancy concentration (~0.026 at.%) can be formed in the Super304H austenitic steel during long-term aging. These ordered Nb-rich carbonitrides are considered to have a MX superstructure based on the fact that they show an ordered structure and the average ratio of metal to non-metal atoms is very close to the stoichiometric MX phase.

## 5. Conclusions

Two types of Super304H steel samples, i.e., solution-treated and long-term (e.g., 5000 h) aged, have been investigated via the techniques of TEM and APT. The results showed that the primary carbonitrides in the Super304H steel are Nb-rich non-stoichiometric compounds with a NaCl-type structure. The crystal structure of the Nb-rich carbonitrides is in a disordered state in the solution-treated state. Interestingly, there is a disorder–order structural transition occurring in the primary carbonitrides in the Super304H steel during long-term aging accompanied by the carbon diffusion from the primary carbonitrides into the austenitic matrix. Based on the current experimental results as well as the thermodynamic and kinetic analysis, the ordering phenomenon in the primary carbonitrides is more likely to be attributed to the long-range ordering of structure vacancies in the non-metal lattice during aging. This is the first time ordered Nb-rich carbonitrides with a MX superstructure at a relatively low structure vacancy concentration (~0.026 at.%) have been revealed, and thus it provides new insights for the understanding of ordering in non-stoichiometric compounds.

## Figures and Tables

**Table 1:** Chemical composition of the Super304H steel (in wt.%).

C	Mn	P	S	Si	Ni	Cr	Cu	Nb	N	Al	B	Fe
0.08	0.787	0.022	0.001	0.29	8.88	17.98	3.066	0.58	0.07	0.012	0.0026	Bal.
